# 

**Published:** 1909-06-19

**Authors:** 


### GRADUATES' MEDICAL SCHOOLS.

DIARY FOR THE WEEK, JUNE 21 to JUNE 26.

### LONDON SCHOOL OF CLINICAL MEDICINE, Seamen's Hospital, Greenwich, S.E.

**At 2.15 p.m.**
**June 23,** Dr. F. Taylor, **Relapsing Fever.**
**At 3.15 p.m.**
**June 25,** Mr. McGavin, **Congenital Malposition of the Testis.**

### THE POST-GRADUATE COLLEGE, West London Hospital, Hammersmith, W.

**At 10 a.m.**
**June 21 and 24,** Surgical Registrar, **Demonstration.**
**June 25,** Medical Registrar, **Demonstration.**
**At 12 noon.**
**June 21,** Dr. Bernstein, **Demonstration.**
**At 12.15 p.m.**
**June 22,** Dr. Pritchard, **Practical Medicine.**
**June 23 and 26,** Dr. Grainger Stewart, **Practical Medicine.**
**At 5 p.m.**
**June 21,** Mr. Bidwell, **Practical Surgery.**
**June 22,** Mr. Bidwell, **Surgical Treatment of Acute General Peritonitis.**
**June 23,** Dr. Beddard, **Medicine—V.**
**June 24,** Dr. Cole, **The Insane and the Law.**
**June 25,** Mr. Lloyd, **Anæsthetics.**

### MEDICAL GRADUATES' COLLEGE AND POLYCLINIC, 22 Chenies Street, W.C.

**At 4 p.m.**
**June 21,** Dr. J. J. Pringle, **Skin.**
**June 22,** Dr. Harry Campbell, **Medical.**
**June 23,** Mr. Lockhart Mummery, **Surgical.**
**June 24,** Sir Jonathan Hutchinson, **Surgical.**
**June 25,** Dr. St. Clair Thomson, **Ear, Nose, and Throat.**
**At 5.15 p.m.**
**June 21,** Dr. Harry Campbell, **Treatment of Asthma.**
**June 22,** Dr. T. D. Lister, **Infantile Scurvy.**
**June 23,** Dr. A. E. Giles, **The Diagnosis of Pelvic Tumours.**
**June 24,** Dr. Theo. Hyslop, **Paralysis and Insanity.**

### NORTH-EAST LONDON POST-GRADUATE COLLEGE, Prince of Wales's General Hospital, Tottenham, N.

**At 4.30 p.m.**
**June 22,** Dr. J. E. Squire, **Demonstration** (at the Northwood Sanatorium).
**June 24,** Dr. A. J. Whiting, **Demonstration of Medical Cases.**

### CENTRAL LONDON THROAT AND EAR HOSPITAL, Gray's Inn Road, W.C.

**At 3.45 p.m.**
**June 22,** Mr. Stuart Low, **The Accessory Sinuses.**

### NATIONAL HOSPITAL FOR THE PARALYSED AND EPILEPTIC, Queen Sq., Bloomsbury, W.C.

**At 3.30 p.m.**
**June 22.** Dr. T. Grainger Stewart, **Nervous Manifestations at the Menopause.**
**June 25.** Dr. T. Grainger Stewart, **Spinal Tumors.**

### THE THROAT HOSPITAL, Golden Square, W.

**At 5.30 p.m.**
**June 21,** Mr. Rose, **Intracranial Complications of Ear Disease Treatment.**

### THE HOSPITAL FOR SICK CHILDREN, Great Ormond Street, W.C.

**At 4 p.m.**
**June 24,** Mr. Fairbank, **Spinal Curvature.**

### BURDETT'S HOSPITALS AND CHARITIES 1909.

By Sir Henry Burdett, K.C.B., K.C.V.O.
(London: The Scientific Press, Ltd. Price 7s. 6d. net. Twentieth Issue.)

There comes a time when certain of the best annual works of reference reach such a pitch of organised efficiency and reliability, that one can hardly imagine how the world ever got on without them. Into this class "Burdett's Hospitals and Charities" made its entry some years ago; but, old friend as it is, there seems to be no limit to the industry, enterprise and originality of its compilers. Every year sees the old features ripened and mellowed, with new ones added; and every year the value of the annual as a work of reference becomes more pronounced. Where improvement seems scarcely possible, each year yet shows something modified in the direction of greater clearness, or some simplification of the difficult subject of hospital finance. A special feature of this year-book, is the lucid manner in which the complicated problems of hospital economics are reduced to order and made easily understandable of the uninitiated. Those who are already familiar with the annual will find herein an advance on previous issues: those who are not may be assured that if ever they want information about any question of hospital and charitable enterprise in any part of the world, it is to this guide-book they may confidently turn for information. Finally, it should be noted that the persistent fallacy which may be summarised in the sentence "The average total annual volume of charitable contributions is fixed and immutable," is once again exposed and refuted.

The Orient Line announce that their new 12,000 ton steamers just built for Australian mail service, are available for a sea-trip holiday to Gibraltar, Marseilles, and the Moorish cities of Spain or Africa. The new steamers are most luxurious, and one of them, the *Otranto*, and the royal yacht H.M.S. *Ophir* will make a series of pleasure cruises to the fjords of Norway. Full particulars can be had on application.

